# Factors Related to Anemia Prevalence Among Women of Childbearing Age in the Period of Global Pandemic

**DOI:** 10.7759/cureus.38491

**Published:** 2023-05-03

**Authors:** Muhammad Hammad, Sadaf Fardoos, Rasikh Arif, Ali Zeb Khan, Anmol Rasheed

**Affiliations:** 1 Pharmacy, Shifa Tameer-E-Millat University, Shifa College of Pharmaceutical Sciences, Islamabad, PAK; 2 Riphah Institute of Pharmaceutical Sciences, Riphah International University, Islamabad, PAK; 3 Al-Shifa Research Centre, Al-Shifa Trust Eye Hospital, Rawalpindi, PAK; 4 Clinical Research, Al-Shifa Trust Eye Hospital, Rawalpindi, PAK; 5 Riphah School of Leadership, Riphah International University, Rawalpindi, PAK

**Keywords:** pakistan, prevalence, women of childbearing age, related factors, anemia, covid-19

## Abstract

Background

Health authorities in Pakistan in collaboration with local and foreign non-government organizations are working hard to achieve the targets set by World Health Organization in 2012, that is, to reduce anemia prevalence and its related factors. However, due to the prevailing COVID-19 crisis, all resources and attention were diverted toward it, which led to ignorance of existing basic health issues.

Objective

This study assesses anemia prevalence and its related factors among women of childbearing age in the period of global pandemic.

Methods

A time-lagged, cross-sectional survey was conducted using a self-administered questionnaire among 1,702 volunteer women aged between 15 and 49 years across five major cities of Pakistan from January 2021 to December 2021 using the non-probability consecutive sampling technique. Blood sample results were analyzed to determine prevalence and anemia severity. The chi-squared test and multiple logistic regression were performed to examine the relationship and effect of related factors with hemoglobin levels using SPSS version 26.

Results

Among the 1,702 respondents, 788 (46.3%) were non-anemic and 914 (53.7%) were anemic. Anemia prevalence in Karachi was slightly greater (n=294, 55.48%) compared to other cities, and the mean hemoglobin level was 11.98 ± 0.92 g/dL. The chi-square test and multiple logistic regression indicated that the respondents’ employment status, mother’s profession, family income, living conditions, chronic health conditions, use of iron and folic acid supplements, junk food, source of drinking water, and knowledge about anemia and its preventive measures were associated significantly with anemia during the pandemic.

Conclusion

Results confirmed that anemia is a multi-factor health problem and that it was totally ignored during the COVID-19 pandemic, as the prevalence increased during the pandemic. Therefore, more attention should be paid to anemia surveillance, anemia awareness programs, and mobilization of community health workers and volunteers to reach a wide range of the population, including women of childbearing age even during the pandemic.

## Introduction

Iron is one of the essential minerals that our bodies require to perform some of its fundamental activities. Iron, as part of structural component of hemoglobin, plays an important role in the transportation of oxygen from the lungs to the body cells [1]. Iron is essential to produce hemoglobin by erythroblasts; thus, if there is an insufficient supply of iron, the cells fail to produce hemoglobin, resulting in a condition known as “anemia” [2]. The normal level of hemoglobin for non-pregnant women and males (aged 15 years and above) is 120 gm/L and 130 gm/L respectively, according to the World Health Organization. If the level is detected below the normal range, we call the condition as “anemia” [2-3].

In the past few decades, anemia has been one of the major global developmental health problems, and this non-communicable disease is increasing rapidly in both developed and developing countries [4]. However, the prevalence of anemia is much higher in underdeveloped countries than in developed countries due to low socioeconomic status and limited healthcare facilities [5-6]. In Pakistan, anemia is categorized as a major nutritional deficiency disorder among infants, pregnant women, and non-pregnant women [6-7]. The COVID-19 pandemic further deteriorated healthcare system and economies of the developing countries, which led to ignorance of existing basic health issues [8].

Almost two billion people across the globe are affected by anemia, either directly or indirectly. The common effects are as follows: delayed normal motor function in infants, increasing risk of low birth weight or premature babies, fatigue in adults, effect on memory, and poor academic performance [9]. An anemic person shows different signs and symptoms, but the common signs and symptoms are chronic fatigue, loss of appetite, headaches, irritability, and loss of concentration [10-11].

Various studies have been conducted among women of different ages across the globe, but no study has been conducted to date that highlights anemia and its related factors during the global pandemic. Moreover, factors such as healthcare utilization, chronic health conditions, junk food, use of iron and folic acid supplements, and availability of clean drinking water have not been studied yet. The current study aims to evaluate the prevalence of anemia in women of childbearing age and to determine the factors linked with it during the global pandemic. The results of the study can help plan nutritional intervention and address the causes that lead to anemia among women of childbearing age in the period of global pandemic.

## Materials and methods

A time-lagged, cross-sectional survey was carried out using a self-administered questionnaire in five major cities of Pakistan (Peshawar, Islamabad, Rawalpindi, Lahore, and Karachi) to study anemia prevalence and its associated characteristics among women of childbearing age between 15 and 49 years from January 2021 to December 2021. Factors such as age, education level, marital status, employment status, family size, father's profession, mother's profession, family income, BMI, menstrual period duration, healthcare utilization, exercise habits, chronic health condition, knowledge about anemia, use of iron and folic acid supplements, use of multi-vitamins, tobacco use, number of meal per day, knowledge about anemia causes, fertility status, breastfeeding, and knowledge about preventive measures were part of the designed questionnaire. The ethical clearance of the study was taken from the Institutional Review Board of Riphah International University, Rawalpindi, Pakistan (Reference no. FMS/RSL/ERC.009). A total of 1,702 respondents’ blood samples were taken, and the questionnaires were distributed after orally explaining to them the purpose of the study and after taking written informed consent from them. Respondents with cognitive or perceptive problems were excluded from the study. The data were collected, compiled, and analyzed using the SPSS (Statistical Package for the Social Science) Version 26 (IBM Corp. Armonk, NY). The blood sample results were used for classification of anemia severity. The independent variable of the study was all related factors, and the dependent variable was hemoglobin levels in women of childbearing age. Descriptive statistics was used to describe the study population characteristics. The chi-square test was used to assess the relationship between associated characteristics and hemoglobin levels, whereas multiple logistic regression analysis was used to assess the individual factors affecting hemoglobin levels. A p-value of ≤0.05 was considered statistically significant.

## Results

Table 1 shows the results of anemia severity in women of childbearing age based on hemoglobin levels. Among the respondents, 788 (46.3%) were non-anemic and 914 (53.7%) were suffering from anemia. Among the 914 (53.6%) respondents with anemia, the prevalence of mild, moderate, and severe anemia was 493 (29.0%), 342 (20.1%), and 79 (4.9%) respectively. The sum of mean hemoglobin levels among all women of childbearing age was 11.98 ± 0.92 g/dL.

**Table 1:** Results of anemia severity in women of childbearing age based on the hemoglobin levels.

Anemia Severity	Frequency (n)	Percent (%)	Mean ± SD (g/dL)
Normal	788	46.4	13.05 ± 0.54
Mild	493	29.0	11.33 ± 0.25
Moderate	342	20.1	9.95 ± 0.47
Severe	79	4.9	7.12 ± 0.12
Total	1,702	100.0	11.98 ± 0.92

Table 2 shows the prevalence of anemia and its level of severity in different cities of Pakistan. Results showed that the prevalence of anemia in Karachi was slightly greater (n=294, 55.48%) compare with that in Peshawar (n=209, 55.00%) and Rawalpindi and Islamabad (n= 211, 54.42%). However, the prevalence of non-anemic respondents was significantly higher in Lahore (n= 205, 50.62%) than other cities. Moreover, the number of severe anemic women was much higher in Karachi (n= 36, 6.78%) compared to other cities.

**Table 2:** Prevalence of anemia and their levels of severity in different cities of Pakistan.

Anemia Severity	Peshawar		Rawalpindi and Islamabad		Lahore		Karachi		Total	
	n	%	n	%	n	%	n	%	n	%
Normal	171	45.00	176	45.48	205	50.62	236	44.52	788	46.30
Mild	110	28.95	121	31.27	89	21.98	173	33.64	493	29.00
Moderate	86	22.63	71	18.34	100	24.68	85	16.03	342	20.10
Severe	13	3.42	19	4.91	11	2.72	36	6.78	79	4.90
Total	380	100.00	387	100.00	405	100.00	530	100.00	1702	100.00

Table 3 shows the results of the chi-square test to find the relationship between associated factors and hemoglobin levels. Of the total 1,702 women, the majority of the women belong to the age group of 31-49 years (41.71%) and 15.15% of them were obese. Also, 50% of them were unmarried and had intermediate education (30.43%); 52.41% of women were unemployed and 34.61% of them had a family income of greater than 100,000. Around 9.93% of them lived in slums and 48.88% of them had knowledge about anemia causes. Most of the women (75.21%) were fecund, 29.14% were using contraception pills, 20.98% breastfed their babies, 77.56% had a one- to two-year gap between their babies, and 91.95% of them lived in urban areas. Moreover, 19.62% and 17.51% of women used iron and folic acid supplements, respectively. The majority of them (90.83%) were non-smokers and had two meals a day, 46.83% and 54.76% drank tap or government supply water or tanker, respectively, 29.20 of them had other chronic health conditions, and 66.86% of them visited healthcare facilities only at the time of illness. Around 25.92% were having two or three children and 35.84% had a family size of seven and more; 55.17% of the women were housewives and 29.91% women’s husbands or fathers were working in the private sector. Around 94.07% have not had any adverse pregnancy outcome, whereas 28.43% had a menstrual period duration of more than five days. The chi-square test showed that age, educational level, marital status, employment status, family size, mother’s profession, family income, menstrual period duration, healthcare utilization, exercise habits, chronic health conditions, living condition, iron supplements, folic acid supplements, junk food, tobacco use, number of meal per day, knowledge about anemia and preventive measures, fertility status, breastfeeding, place of residence, drinking water source, husband’s or father’s occupation, number of children, contraception pill use, and adverse pregnancy outcome had a relationship with hemoglobin levels, whereas pregnancy gap, and BMI showed no significant relationship with hemoglobin levels.

**Table 3:** Results of the chi-square test to reveal the factors and their association with hemoglobin levels among women of childbearing age.

Factors	Total (Weighted)	Anemic		Non-Anemic		Total		p-value
		N	%	n	%	n	%	
Age	1702							0.001
15-30		118	12.91	222	28.17	340	19.98	
31-40		353	38.62	299	37.94	652	38.31	
31-49		443	48.47	267	33.89	710	41.71	
Educational level	1702							0.001
Illiterate		291	31.84	101	12.82	392	23.03	
Matric		240	26.26	145	18.40	385	22.62	
Intermediate		248	27.13	270	34.26	518	30.43	
Bachelor& above		135	14.77	272	34.52	407	23.92	
Marital status	1702							0.001
Married		337	36.87	433	54.95	770	45.24	
Unmarried		515	56.35	336	42.64	851	50.00	
Divorce		62	6.78	19	2.41	81	4.76	
Employment status	1702							0.001
Employed		448	49.02	362	45.94	810	47.59	
Unemployed		466	50.98	426	54.06	892	52.41	
Family size	1702							0.001
≤ 4		101	11.05	147	18.65	248	14.57	
5-6		493	53.94	351	44.54	844	49.59	
≥ 7		320	35.01	290	36.81	610	35.84	
Mother profession	1702							0.001
House wife		508	55.58	431	54.70	939	55.17	
Government employee		123	13.46	118	14.97	241	14.16	
Private sector		145	15.86	132	16.74	277	16.27	
Business		119	21.77	90	11.42	209	12.28	
Others		19	2.08	17	2.16	36	2.12	
Family income	1702							0.001
≤ 20000		195	21.33	85	10.79	280	16.45	
20001-50000		110	12.04	213	27.03	323	18.98	
50001-99999		247	27.02	263	33.38	510	29.96	
≥ 100000		362	39.61	227	28.80	589	34.61	
BMI	1702							0.180
Underweight		238	26.04	111	14.09	349	20.51	
Normal		351	38.40	476	60.41	827	48.59	
Overweight		165	18.05	103	13.07	268	15.75	
Obese		160	17.51	98	12.43	258	15.15	
Menstrual period duration	1702							0.001
No		108	11.82	56	7.11	164	9.64	
2-3 days		170	18.59	170	21.57	340	19.98	
4-5 days		242	26.48	472	59.90	714	41.95	
>5 days		394	43.11	90	11.42	484	28.43	
Healthcare utilization	1702							0.001
Only in illness		566	61.93	572	72.59	1138	66.86	
1-2 years		276	30.19	165	20.94	441	25.91	
Every 6 months		72	7.88	51	6.47	123	7.23	
Exercise habits	1702							0.001
Not at all		492	53.83	453	57.49	945	55.52	
Sometimes		288	31.51	243	30.84	531	31.20	
Daily		134	14.66	92	11.67	226	13.28	
Chronic health condition	1702							0.001
Yes		306	33.48	191	24.24	497	29.20	
No		608	66.52	597	75.76	1205	70.80	
Living conditions	1702							0.001
Normal house/Flat		768	84.03	765	97.08	1533	90.07	
Slums		146	15.97	23	2.92	169	9.93	
Iron supplements use	1702							0.001
Yes		134	14.66	200	25.38	334	19.62	
No		780	85.34	588	74.62	1368	80.38	
Folic acid supplements	1702							0.022
Yes		104	11.38	194	24.62	298	17.51	
No		810	88.62	594	75.38	1404	82.49	
Junk food	1702							0.001
Mostly		519	56.78	335	42.51	854	50.18	
Rarely		395	43.22	453	57.49	848	49.82	
Tobacco use	1702							0.001
Yes		73	7.99	83	10.53	156	9.17	
No		841	92.01	705	89.47	1546	90.83	
No of meals per day	1702							0.001
1		85	9.30	45	5.71	130	7.64	
2		414	45.30	383	48.60	797	46.83	
≥ 3		415	45.40	360	45.69	775	45.53	
Knowledge about anemia causes	1702							0.001
Yes		383	41.90	449	56.98	832	48.88	
No		531	58.10	339	43.02	870	51.12	
Preventive measure knowledge	1702							0.001
Yes		377	41.25	530	67.26	907	53.29	
No		537	58.75	258	32.74	795	46.71	
Fertility status	1702							0.001
In fecund		55	6.01	140	17.77	195	11.46	
Fecund		702	76.81	578	73.35	1280	75.21	
Pregnant		54	5.91	25	3.17	79	4.63	
Post-partum amenorrhea		103	11.27	45	5.71	148	8.70	
Breastfeeding	1702							0.001
Yes		227	24.84	130	16.50	357	20.98	
No		687	75.16	658	83.50	1345	79.02	
Place of residence	1702							0.001
Rural		58	6.35	79	10.03	137	8.05%	
Urban		856	93.65	709	89.97	1565	91.95%	
Husband or Father occupation	1702							0.001
Government employee		230	25.16	132	16.75	362	21.27	
Private sector		268	29.33	241	30.59	509	29.91	
Business		274	29.98	189	23.98	463	27.20	
Skilled worker		128	14.00	162	20.56	290	17.04	
Agriculture		14	1.53	64	8.12	78	4.58	
Drinking water source	1702							0.035
Tap or Government supply or Tanker		553	60.50	379	48.10	932	54.76	
Well		201	21.99	285	36.16	486	28.55	
Others (Mineral water)		160	17.51	124	15.74	284	16.69	
No of children	1702							0.001
0		368	40.26	488	61.93	856	50.29	
1-2		126	13.79	96	12.18	222	13.04	
2-3		289	31.62	152	19.30	441	25.92	
> 3		131	14.33	52	6.59	183	10.75	
Adverse pregnancy outcome	1702							0.001
No		833	91.14	768	97.46	1601	94.07	
Yes (stillbirth, miscarriage, abortion)		81	8.86	20	2.54	101	5.93	
Contraception pill	1702							0.001
Yes		318	34.79	178	22.59	496	29.14	
No (others)		596	65.21	610	77.41	1206	70.86	
Pregnancy gap	1702							0.735
0 years		56	6.13	78	9.90	134	7.87	
1-2 years		735	80.42	585	74.24	1320	77.56	
3-4 years		3	13.45	125	15.86	248	14.57	

The computed results of multiple logistic regression analysis in Table 4 showed that BMI, family size, healthcare utilization, exercise habits, tobacco use, number of meals per day, and place of residence did not show a significant association with hemoglobin levels, whereas education level, employment status, chronic health conditions, junk food, anemia cause knowledge, preventive measure knowledge, breastfeeding, husband’s or father's occupation, contraception use, and repeated pregnancy have a negative association with hemoglobin levels. On the other hand, age, source of drinking water, marital status, mother's profession, family income, menstrual period duration, living conditions, iron supplement use, folic acid use, fertility status, number of children, and adverse pregnancy outcome have a positive impact on hemoglobin levels among women of childbearing age.

**Table 4:** Results of multiple logistic regression to reveal the effect of related factors on hemoglobin levels.

Associated Characteristics	B	df	95% CI		P-value
			Lower	Upper	
Age	0.083	2	0.060	0.105	0.001
Educational level	-0.091	3	-0.107	-0.075	0.001
Marital status	0.091	2	0.035	0.147	0.001
Employment status	-0.078	1	-1.33	-0.023	0.005
Family size	-0.006	2	-0.030	0.019	0.637
Mother’s profession	0.051	4	0.034	0.067	0.001
Family income	0.028	3	0.000	0.056	0.043
BMI	-0.005	3	-0.022	0.012	0.580
Menstrual period duration	0.051	3	0.026	0.077	0.001
Healthcare utilization	0.010	2	-0.030	0.051	0.619
Exercise habits	0.001	2	-0.046	0.048	0.973
Chronic health condition	-0.408	1	-0.467	-0.349	0.001
Living conditions	0.657	1	0.574	0.740	0.001
Iron supplement use	0.241	1	0.138	0.345	0.001
Folic acid supplement use	0.353	1	0.250	0.456	0.001
Junk food	-0.121	1	-0.171	-0.071	0.001
Tobacco use	0.045	1	-0.034	0.125	0.263
Number of meals per day	-0.056	2	-0.119	0.006	0.074
Knowledge about anemia causes	-0.263	1	-0.338	-0.188	0.001
Preventive measures knowledge	-0.260	1	-0.309	-0.211	0.001
Fertility status	0.049	3	0.026	0.073	0.001
Breastfeeding	-0.053	1	-0.096	-0.010	0.017
Place of residence	-0.010	1	-0.086	0.067	0.803
Husband’s or father’s occupation	-0.096	4	-0.116	-0.076	0.001
Drinking water source	0.024	2	-0.010	0.058	0.0162
Number of children	0.058	3	0.034	0.083	0.001
Adverse pregnancy outcome	0.247	1	0.173	0.321	0.001
Contraception pill	-0.120	1	-0.168	-0.072	0.001
Pregnancy gap	-0.121	2	-0.154	-0.089	0.001

## Discussion

Anemia is the most common nutritional deficiency disorder among infants, pregnant women, and non-pregnant women [6]. The targets and action plans were designed and approved by the World Health Organization for reducing the prevalence of anemia [12-13]. The current study was designed to reveal the factors that influence anemia and its prevalence among women of childbearing age in Pakistan during this period of the COVID-19 pandemic.

The results from this study showed total anemic respondents to be greater (53.7%) than non-anemic respondents (46.3%). The results from previous studies were in accord with the present findings, but the prevalence of anemia increased during the pandemic [9]. Around half a million women of reproductive age are anemic worldwide, with a higher burden of anemia in low- and middle-income countries. Specifically, the maximum of anemia burden lies in Southeast Asian countries [10]. This study also indicated severe anemia to be present in 4.9% of women of childbearing age. A long-term vitamin and mineral deficiency, especially of iron, can lead toward anemia [14]. Anemia, especially iron deficiency anemia, progresses gradually usually without any signs and symptoms until anemia attains severity [15]. The mean hemoglobin level reported in this study was 11.98 ± 0.92 g/dL, which is in close accord with previous findings (12.35 ± 1.80 g/dL) [16]. Table 2 shows the citywise distribution of anemia in Pakistan. Karachi is the largest city with anemia prevalence (n=294, 55.48%) followed by Peshawar (n=209, 55.00%) and Rawalpindi and Islamabad (n= 211, 54.42%). Previous studies conducted in Agha Khan University also reported similar findings [17]. While the prevalence of anemia was reported to be low in this study, a previous study conducted in Lahore reported low income to be the most significant factor of anemia presence [18]. Therefore, a greater understanding of the various determining factors that can potentially lead towards anemia is of great importance for local investors to effectively resolve this issue [19].

Table 3 presents the results of the chi-square test to reveal the relationship between associated factors and hemoglobin levels, while Table 4 shows results of multiple logistic regression to reveal the effect of associated characteristics on hemoglobin levels. All the other factors showed a significant relationship with hemoglobin levels similar to the ones revealed by the chi-square test except for BMI, healthcare utilization, family size, exercising habits, tobacco use, number of meals per day, and place of residence, which all had an insignificant impact on hemoglobin levels. The results showed age to have a significant relationship with hemoglobin levels. The World Health Organization estimated that the majority (56%) of pregnant and women of childbearing age were anemic. Iron deficiency at this age is thought to be caused by a multitude of factors such as previously decreased iron supplies, growing requirement of the fetus, and increased blood volume [20]. Level of education was also significantly associated with hemoglobin levels. Previous studies conducted in this regard reported contradictory findings. In a study of low- and middle-income countries, education levels along with cultural norms were a major determinant of anemia severity [21]. Another study suggested level of schooling to not be the determining factor for anemia in Nepal and Pakistan, whereas it was inverse for countries like India and Bangladesh where there existed a relationship between the two factors [22]. Moreover, the results of this study regarding family income, fathers' and mothers’ profession, and family size were also in accord with previous findings that higher monthly income of both parents was associated with a lower occurrence of anemia [9]. Similarly, menstrual period duration and exercise habits were also significantly related to anemia. According to Kamruzzaman et al., high menstrual bleeding decreases iron levels and hemoglobin amount, leading to severe anemia if not treated [23]. The results of this study also indicated economic status and living conditions to be significantly associated with hemoglobin levels, which is also in agreement with previous findings of studies from different parts of the world and in Pakistan [24]. Moreover, the prevalence of anemia was higher among women whose mothers were housewives as compared with government employee or businesspersons. Similarly, the prevalence was low among women with fathers or husbands who were government employees, and these findings were synchronous with the findings of previous studies [25].

As this study indicated that anemia has a strong association with chronic health conditions, such as infections, autoimmune diseases, cancer, and chronic kidney disease, it is a matter of great concern to prevent such chronic conditions; the results were coeval with previous findings [24]. The use of supplements especially iron supplement was significantly associated with hemoglobin level. Therefore, all women of reproductive age should take supplements especially of iron to prevent anemia [26]. The past literature suggested junk food consumption and smoking to be associated with anemia presence. Factors such as consuming tea and coffee after meals, not using iron supplements, decreased consumption of vegetables, and more emphasis on junk food were linked to anemia severity [27]. Similarly, the presence of anemia was significantly higher in smokers and opium users, which is in accord with the present findings [28]. Previous literature also suggested that receiving antenatal and perinatal care and proper follow-ups was associated with decreased likelihood of anemia. Anemia was much higher for mothers with less or no antenatal care [29]. Similarly, the number of children in the family, meals consumed in a day, contraception use, and adverse pregnancy outcomes were all associated with anemia presence. As there is a need to feed more children, the demand for food will increase, leading to poor quality of care and a greater anemia risk [25]. The risk was also greater among meal skippers or people who consumed a smaller number of meals a day. It was reported that meal frequency of less than two meals a day was found to be associated with anemia presence in pregnant women [1]. Anemia in the third trimester of pregnancy is associated with adverse maternal and neonatal outcomes. A retrospective study from Pakistan reported both adverse maternal and neonatal outcomes such as postpartum hemorrhage, prolonged or obstructed labor, gestational hypertension, and preeclampsia, with neonatal complications ranging from early neonatal death, stillbirth, preterm delivery, to low birth weight [2]. Moreover, anemia risk in urban women is reduced up to 73% due to better health and sanitation facilities [3]. Similarly, the study indicated a strong association of living conditions and source of drinking water, with hemoglobin levels, as women living in houses with unhygienic toilet facility and source of drinking water were more likely to develop anemia as they could be more prone to both water- and food-borne infections such as helminthic infections such as hookworm, which might, in turn, increase anemia severity [4]. While the results revealed some significant factors, BMI and pregnancy gap had no significant association with hemoglobin levels. A study conducted by Habib et al. found no significant association between anemia and BMI [5]. Another study suggested odds of anemia decrease with obesity [7]. The difference in results between studies might be due to difference in age and geographic location of the study participants, as suggested by Hess et al. [1]. The present study findings suggested an insignificant relationship between pregnancy gap and hemoglobin levels, which is in contradiction to previous studies. A systematic review on effect of birth spacing on maternal and child health revealed that short birth intervals were associated with maternal anemia [8]. Women who had less than two years between pregnancies were more likely to be anemic this can be justified by the fact that such women got less time to recover from depleted nutrients, thereby increasing anemia severity [3].

Lastly, the results of this study were convergent with previous studies that indicated that family income [19], exercise habits [10], number of meals [24], and education levels [11] were the societal determinants of anemia among women of reproductive age. Awareness about appropriate age for marriage, education status, employment opportunities, healthcare utilization, avoidance of excessive blood loss, provision of iron and folic acid supplements, adverse, junk food, and family planning should be imparted to masses especially in slums and rural areas to reduce the prevalence of anemia among women of childbearing age. Furthermore, the results showed that anemia is a multi-factorial health problem, and prevention strategies should focus on addressing these significant determinants for reducing the severity of anemia even during the time of a global pandemic.

Limitations of the study

The current study had a few limitations. First, we had no knowledge of the hemoglobin levels before the pandemic; therefore, we classified anemia based on the current hemoglobin levels as per defined guidelines by the World Health Organization. Second, we did not assess serum iron, ferritin, or transferrin levels, which are further anemia indicators. Third, factors such as cultural background, domestic violence, and drug abuse have not been studied.

## Conclusions

The current study illustrates the risk factors for anemia among women of childbearing age in Pakistan during the COVID-19 pandemic. It is evident from the current findings that significant improvement should need to be made to further improve the overall health status of women. The prevalence of anemia is 53.7% according to this study, which was significantly more than the previous study conducted before the COVID-19 pandemic. The results also confirmed that anemia is a multi-factor health problem and was ignored during the pandemic. It was also found that awareness about the appropriate age for marriage, education status, employment opportunities, clean drinking water, healthcare utilization, avoidance of excessive blood loss, provision of iron and folic acid supplements, junk food, and family planning should be imparted to masses especially in slums and rural areas to reduce the anemia prevalence; therefore, more attention should be given to anemia surveillance and awareness programs, as well as mobilization of community health workers and volunteers to reach a wide section of the populace, including women of childbearing age during the times of pandemic.
